# Primary care for tinnitus: practice and opinion among GPs in England

**DOI:** 10.1111/j.1365-2753.2011.01696.x

**Published:** 2011-08

**Authors:** Suliman K El-Shunnar, Derek J Hoare, Sandra Smith, Phillip E Gander, Sujin Kang, Kathryn Fackrell, Deborah A Hall

**Affiliations:** 1 Core Trainee 2 (ENT), Ear, Nose and Throat Department, Queens Medical Centre, Nottingham University Hospitals NHS TrustNottingham, UK; 2 Research Fellow, NIHR National Biomedical Research Unit in Hearing, School of Clinical Sciences, The University of NottinghamRopewalk House, Nottingham, UK; 3 Research Associate, NIHR National Biomedical Research Unit in Hearing, School of Clinical Sciences, The University of NottinghamRopewalk House, Nottingham, UK; 4 Statistician, NIHR National Biomedical Research Unit in Hearing, School of Clinical Sciences, The University of NottinghamRopewalk House, Nottingham, UK; 5 Undergraduate Student, NIHR National Biomedical Research Unit in Hearing, School of Clinical Sciences, The University of NottinghamRopewalk House, Nottingham, UK; 6 Scientific Director, NIHR National Biomedical Research Unit in Hearing, School of Clinical Sciences, The University of NottinghamRopewalk House, Nottingham, UK and Professor in Cognitive Neuroscience, Division of Psychology, School of Social Sciences, Nottingham Trent UniversityNottingham, UK

**Keywords:** Department of Health, Good Practice Guidelines, GP education

## Abstract

**Rationale, aim and objective:**

Effective tinnitus management starts with appropriate general practitioner (GP) triage, which in England can be guided by the Department of Health's Good Practice Guide (GPG). Despite the prevalence of the condition, there has never been a systematic survey of its management in primary care in England. We aimed to evaluate how people with tinnitus are assessed and managed in general practice, noting variation in practice across GPs and health authorities, and evaluating how closely typical practice aligns to the GPG for tinnitus.

**Methods:**

A nine-item postal questionnaire was sent to 2000 GPs randomly selected to proportionally represent the number of primary care trusts and strategic health authorities in England.

**Results:**

We received 368 responses. Responses indicated a mix of frequent and infrequent practices, for example, 90% of GPs assessed the impact of tinnitus on quality of life, but fewer examined cranial nerves (38%) or assessed for a carotid bruit (26%) during a tinnitus consultation. In the management of tinnitus, 83% routinely removed earwax, and 87% provided information-based advice. In contrast, only 4% of responders would offer antidepressant drugs or psychological therapies. Thematic analysis revealed a desire for concise training on tinnitus management.

**Conclusions:**

GP assessment and management of tinnitus represents potential inequity of service for tinnitus patients. While the GPG aims to promote equity of care, it is only referred to by a minority of clinicians and so its utility for guiding service delivery is questionable. Although some GPs highlighted little demand for tinnitus management within their practice, many others expressed an unmet need for specific and concise GP training on tinnitus management. Further work should therefore evaluate current informational resources and propose effective modes of delivering educational updates.

## Introduction

Tinnitus is defined as the perception of sound in the absence of any corresponding external acoustic energy. Eight per cent of the population will seek medical advice about tinnitus, and some suffer debilitating symptoms such as anxiety, depression or sleep disturbances that have a detrimental impact on their quality of life [1].

There is no singly effective treatment for tinnitus, and so the aim of medical intervention is to manage rather than cure it. Effective management begins with appropriate triage at the primary care level [i.e. the general practitioner (GP)]. Guidance on triage is provided by the Department of Health's Good Practice Guide (GPG) for the provision of services for adults with tinnitus [2]. This document was largely generated from expert opinion and the experiences of clinicians from a broad range of disciplines, including general practice, and provides suggestions for how each level of service should be delivered to provide equity of care to all adults who have troublesome tinnitus. Our recent survey of current audiological services in England, however, highlighted the many challenges to equity of care [3]. In particular, there is no high quality evidence-base for most common tinnitus management strategies or protocols in use [4].

The GPG represents a patient-centred approach to tinnitus management, such that it departs from more medical model guidelines, such as the algorithm from the international Tinnitus Research Initiative working group [5]. For example, the GPG suggests that subsets of tinnitus patients can ‘bypass’ specialist ear, nose and throat (ENT) centres and be referred directly to audiological services, while the Tinnitus Research Initiative recommends that management of *all* tinnitus patients should start with their assessment by a neuro-otological specialist [6–8]. Essentially, the GPG represents a shift towards reduced referral to ENT, increasing the responsibility of GPs and local service audiologists to observe potential indicators of pathologies related to tinnitus which would require specific management by ENT or tertiary neuro-otological specialities. Given this increased responsibility, it is important to ascertain how effectively GPs currently manage or refer their tinnitus patients. Eliciting GP opinion is also timely given the changes in health service commissioning described in the Coalition White Paper *Equity and excellence: Liberating the NHS*, whereby primary care trusts are to be dissolved, with handover of the commissioning of health care services to GP consortia [9]. This could potentiate changes in the services that are commissioned for tinnitus patients.

Here we present responses to a questionnaire that evaluated GP tinnitus management practices, the resources they use, and opinions on what makes for successful tinnitus management. To our knowledge, this is the first systematic national survey of GP tinnitus management in England.

## Participants and methods

### Questionnaire development

A systematic approach to survey design was informed by Kelley *et al*. [10] and Burns *et al*. [11]. First, authors compiled a list of potential items for inclusion in the questionnaire, which were then grouped into topics. The choice of these topics was based on National Health Service (NHS) publications and online resources, scientific papers, and anecdotal comments from clinical colleagues in audiology and ENT. Nine topics emerged: assessment, management, resources, referral pathways, support, tinnitus training, guidelines, GP/patient satisfaction and GP opinions on tinnitus management in primary care. Authors then generated a large number of potential questions based on the items within each topic. Through author discussions, questions within each topic were then distilled down to leave one, generating a potential nine-item questionnaire.

This questionnaire was piloted on seven GP colleagues from the East Midlands and South Central strategic health authorities (SHAs), four of whom have a special interest in ENT. The aim of piloting was to assess the construct validity of the questions and response options, and the face validity of the questions [11]. Feedback was used to refine some of the original questions. The final questionnaire maintained nine questions and is given in Appendix 1. Eight questions had multiple tick boxes, two with an option for further comment. Question 9 was left open to elicit personal opinions and to identify how strongly attitudes were held or not.

### Sample selection

We aimed to elicit responses from 400 to 600 GPs in a single mail-out. Based on an expected response rate of 20–30% [10,12] we selected a random sample of 2000 GPs to receive the questionnaire. Named GPs were selected using two NHS websites (http://www.ic.nhs.uk and http://www.nhs.uk) with selections determined using a random number generator (available at http://www.random.org). Questionnaires were sent to 200 GPs in each of the 10 SHAs in England. Every primary care trust within each SHA was represented and the questionnaire was sent to only one named GP per practice. The survey was mailed on 14 May 2010, and no reminders were sent. A return envelope, covering instruction letter and details of a prize draw for all those responding by 18 June 2010 were included.

### Data analysis

Data (quantitative and free text) were recorded in an entry database (Microsoft Access). Statistical analysis was performed in spss (version 16.0). Free text responses were subjected to a thematic content analysis. Thematic analysis is a method that is widely used in qualitative research [3,13–15]. For full details of the protocol followed here, see Hoare *et al*. [3]. In brief, responses to open questions were analysed independently by pairs of authors in an iterative process of reading, re-reading, and selecting features of individual responses (codes) that appear relevant to the question asked. Codes that were considered equivalent were grouped under ‘proposed themes’. Only after this stage, did those authors analysing text from the same set of responses meet to agree ‘codes’ and ‘proposed themes’, revisiting the full dataset to confirm the likeness of codes within themes and the distinctiveness of codes allocated to different themes. All themes were ultimately agreed by all five authors involved in the thematic analysis.

Data are presented as proportions ±95% confidence intervals, calculation of which were based on our sample size of 368, and an estimated population of 31 000 GPs in England [16].

## Results

### Demographics

From a single mail-out to 2000 GPs we anticipated 400–600 responses and received 368 (18% response rate). We did not have the resources to post a tranche of follow-up letters but responses were received from GPs across all 10 SHAs (from 12% response rates from London and the West Midlands to 22% from the South West). Sixty-five responders (18%) identified themselves as having an interest in ENT. GPs reported running an average of 7.3 clinical sessions per week.

**Figure 1:**
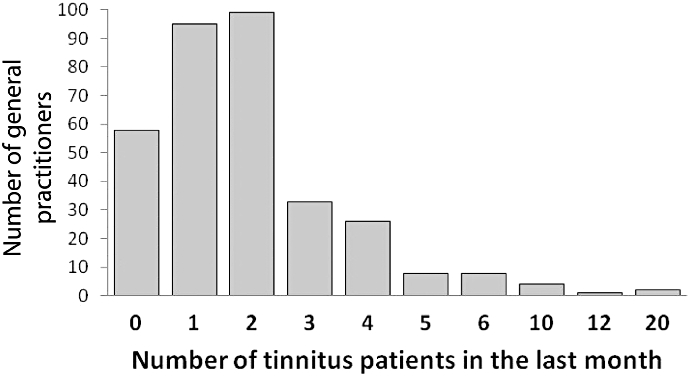
Number of primary tinnitus consultations in the month before completing the questionnaire.

When GPs were asked how many patients they had seen in the last month whose primary complaint was tinnitus, responses varied from none to 20 (Fig. 1). There were no significant differences between the numbers reported from each SHA. The average number of tinnitus consultations per GP per month was two, suggesting that the annual number of such consultations in England is in the region of 0.75 million (based on the previous estimate of 31 000 GPs).

### Knowledge of tinnitus

**Figure 2:**
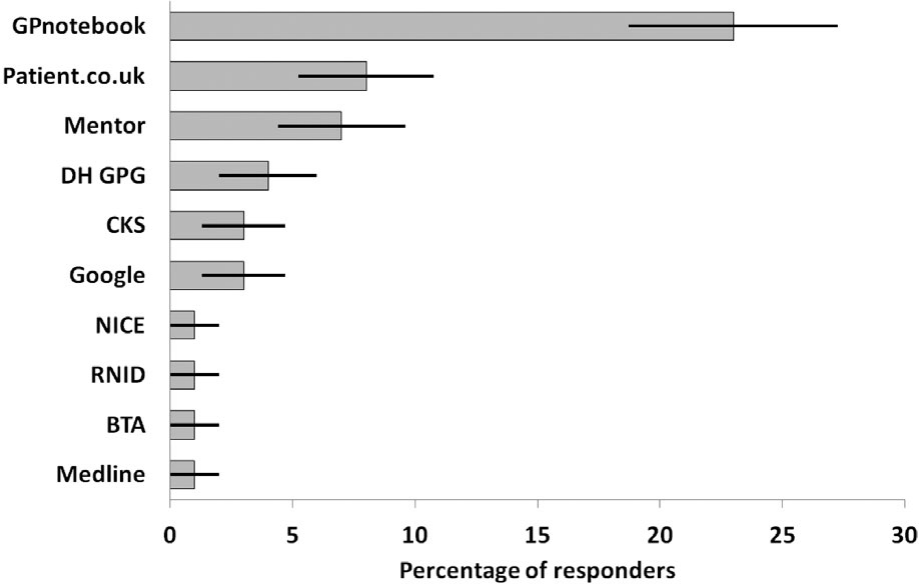
Internet sources of information on tinnitus consulted by general practitioners (GPs). Numbers are number of GPs who indicated each response item ±95% confidence intervals. BTA, British Tinnitus Association; CKS, Clinical Knowledge Summaries; DH GPG, Department of Health Good Practice Guide; NICE, National Institute for health and Clinical Excellence; RNID, Royal National Institute for Deaf People.

When asked if tinnitus was a topic on which they sought information, 266 responders (76%) reported that they did, with 20% looking to a colleague for advice. Only 51 (14%) reported using the GPG, although GPs with an interest in ENT were significantly more likely to refer to the GPG than those without an interest (*P* = 0.01, Pearson's chi-squared test). Fifty-five per cent of responders used the internet as their source of tinnitus information, with GPnotebook (87 GPs, 43%) being the most common (Fig. 2). Only 12 GPs (3%) reported that they access relevant charity websites, such as that of the British Tinnitus Association, as a source of information.

### Assessment and examination

**Figure 3:**
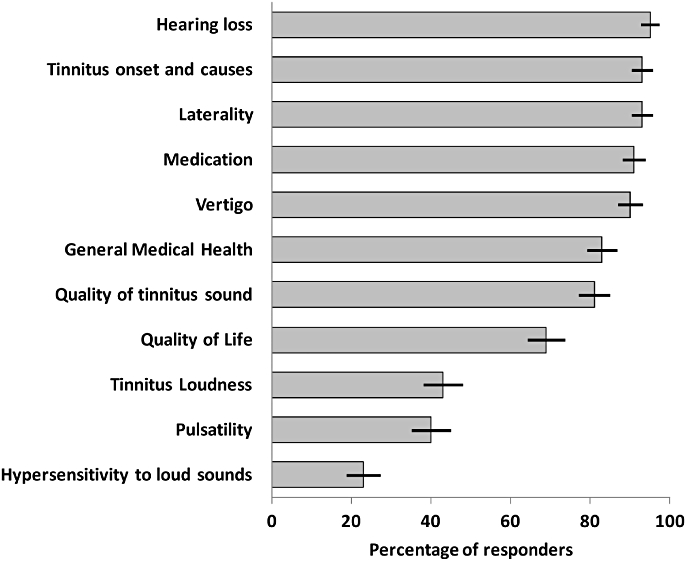
Information routinely obtained when taking a patient history relating to tinnitus. Numbers are number of general practitioners who indicated each response item ±95% confidence intervals.

General practitioners were asked which of 11 history questions they addressed during a tinnitus patient consultation (Appendix 1, Question 1) and which of five examinations they routinely performed (Appendix 1, Question 2). All response options appear in the GPG [2]. On average, eight of the 11 history questions were routinely assessed (Fig. 3). Some aspects, such as tinnitus onset or laterality were assessed by almost all GPs, 95% and 94% of responders respectively. Assessments for tinnitus pulsatility, and hypersensitivity to loud sounds were least common (39% and 23% respectively). There were some examples of geographical variability in the data, for example, responders from the North East SHA reportedly asked significantly less often about tinnitus loudness than GPs from the South West (*P* < 0.01, Fisher–Freeman–Halton test and Fisher's exact test).

General practitioners with an interest in ENT were more likely to ask about pulsatility than GPs who did not have an interest in ENT (*P* = 0.012, Pearson's chi-squared test). This is noteworthy because pulsate tinnitus is likely to have a physical cause, such as hypertension or otitis media, that can be treated medically. Moreover, pulsatile tinnitus is one of the specific recommendations for onward referral to a specialist centre and so should be investigated by the GP.

**Figure 4:**
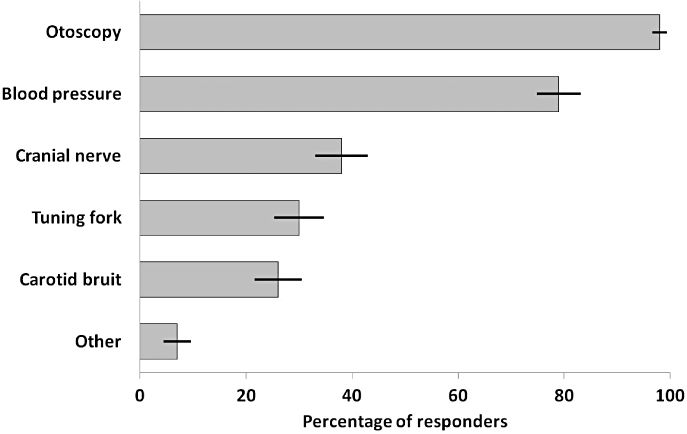
Examinations routinely performed for patients who have tinnitus. Numbers are number of general practitioners who indicated each response item ±95% confidence intervals.

Tinnitus examination is similarly variable across the cohort (Fig. 4). Almost all responders (99%) routinely performed otoscopy, but only 26% of responders routinely listened for a carotid bruit, 38% performed cranial nerve examination and 31% routinely performed a tuning fork test. We did not find any geographical variability in reported use of tinnitus examinations.

### Managing tinnitus in primary care

The GPG suggests that bilateral, mild, non-troublesome tinnitus without hearing difficulty can be managed in primary care through initial advice and reassurance, excluding the existence of wax or external ear infections, or other conditions which may result in tinnitus [2]. It further suggests that GPs may manage tinnitus patients with antidepressants, anxiolytics or night sedation as required.

**Figure 5:**
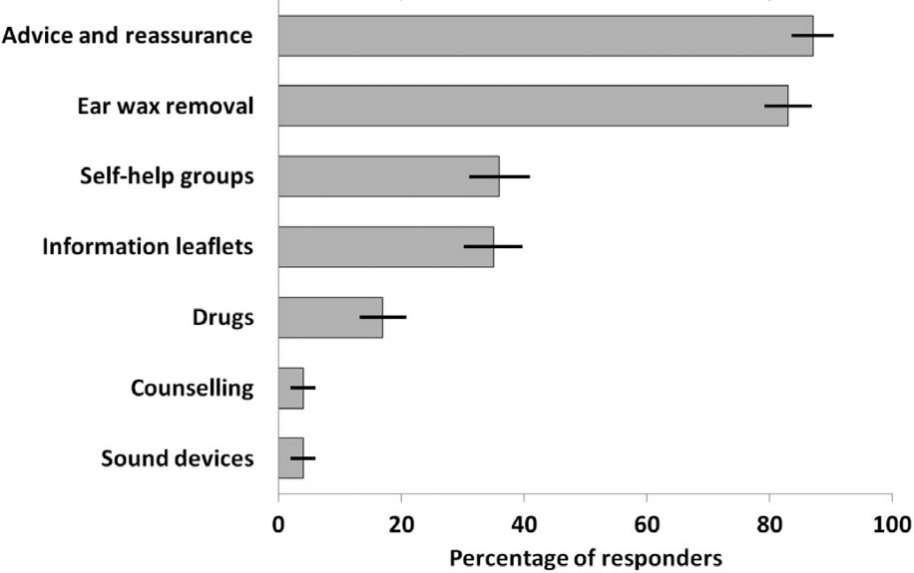
Tinnitus treatments routinely used in general practice. Numbers are number of general practitioners who indicated each response item ±95% confidence intervals.

We asked GPs about the tinnitus management options they routinely offer in their practice (Appendix 1, Question 3). Most responders offered ear-wax removal (83%), and gave advice and reassurance (87%) (Fig. 5), but fewer GPs recommended self-help groups (36%) or provided information leaflets (35%). Seventeen per cent of responders reported that they would prescribe drug therapies as part of their tinnitus management; with 9% prescribing betahistine and 4% prescribing antidepressants. A minority (<3%) of responders reported that they would prescribe prochlorperazine, cinnarizine or beclometasone for tinnitus. Significantly more GPs with an interest in ENT said they would prescribe drug therapies for tinnitus than GPs who did not have an interest in ENT (*P* < 0.001, Pearson's chi-squared test). GPs rarely offered in-house counselling (4%) or sound devices (4%). In fact, no provision of sound devices was reported by responders from the West Midlands, London, South West or the East of England SHA. Significantly more GPs from the North East SHA reported that they provide sound devices than from any other SHA (*P* = 0.04, Fisher–Freeman–Halton test and *P* < 0.01, Fisher's exact test).

### Referring tinnitus patients onwards

The GPG recommends that patients with non-troublesome tinnitus and those with additional hearing difficulty can be referred directly to local community audiology services for management. Patients with tinnitus that is distressing, unilateral or pulsatile, or tinnitus with a suspected associated disorder of medical, otological or psychological origin should be referred to second-level specialist audiology/ENT services and other specialist clinical services as appropriate [2].

When asked about initial onward referral (Appendix 1, Questions 4 and 5) three responders (<1%) said that they *never* referred tinnitus patients onwards, whereas 14 responders (4%) referred *all* of their tinnitus patients onwards. On average, 37% of tinnitus patients were referred, most to ENT (82%) or audiology (12%), or to GP colleagues (5%) (Table 1). A minority said they referred tinnitus patients directly to psychiatry or psychology specialities (less than 1% in both cases). Responders who were part-time, or who did not have an interest in ENT were significantly more likely to refer tinnitus patients onwards than those with an interest in ENT (*P* = 0.04, Mann–Whitney *U*-test). Rates of referral also differed between SHAs (Table 1) but not significantly. Referral rates were highest in the East of England and North West SHAs (43% of patients) and lowest in London (29%). GPs expressed mixed views about following particular criteria to guide their decision about onward referral (Appendix 1, Question 4), 52% said they followed a particular criterion and 48% did not. Nineteen responders who indicated that they did not follow referral criteria further commented that they were not aware of any formal criteria *to* follow. Responders who commented further on this stated that they referred patients who reported unilateral tinnitus (34%), hearing loss (14%) or tinnitus of recent onset (5%). Again, it was interesting to note that pulsatile tinnitus was not mentioned here despite being one of the specific referral criteria recommended by the GPG and by GPnotebook.

**Table 1:** Rate and direction of initial referrals according to strategic health authority

		Of which % referred to:				
		
Strategic health authority	% of total referred	ENT	Audiology	Psychiatry	Psychology	Other GP
East Midlands	38	87	11	<1	<1	0
East of England	43	93	5	<1	<1	<1
London	29	85	4	<1	2	8
North East	40	90	9	<1	<1	0
North West	43	82	12	2	<1	4
South Central	36	69	22	0	0	9
South East	35	82	13	0	<1	5
South West	34	78	17	<1	<1	4
West Midlands	31	68	20	<1	<1	11
Yorkshire & Humber	36	85	8	0	0	7
**Overall mean**	**37**	**82**	**12**	**<1**	**<1**	**5**

ENT, ear, nose and throat; GP, general practitioner.

### Satisfaction and improving tinnitus management in primary care

Using a visual analogue scale from 0 to 10, GPs were asked to rate (i) their level of satisfaction with how they manage tinnitus, and (ii) how satisfied they believed their patients were with the management offered in primary care (Appendix 1, Question 6). Mean satisfaction ratings for each SHA are given in Table 2. The overall mean GP satisfaction rating was 5.9 and the average perceived patient satisfaction rating was 5.4. There was a strong correlation between GP ratings of their own satisfaction and that of their patients (Spearman's *r* = 665 and Kendall's tau = 0.533, *P* < 0.0001). There was a significant higher mean self and patient satisfaction score reported by GPs with an interest in ENT (*P* < 0.001 for both cases, Mann–Whitney *U*-test).

**Table 2:** General practitioner (GP)-rated satisfaction with their tinnitus service (mean per strategic health authority)

Strategic health authority	GP satisfaction	Patient satisfaction
East Midlands	6.1	5.5
East of England	5.8	5.5
London	5.5	5.1
North East	6.3	5.6
North West	6.2	5.5
South Central	5.5	5.6
South East	5.8	5.5
South West	6.3	5.5
West Midlands	5.7	4.5
Yorkshire & Humber	5.9	5.8
**Mean**	**5.9**	**5.4**

Ratings on a scale from 0 to 10 where 10 indicates extreme satisfaction.

**Figure 6:**
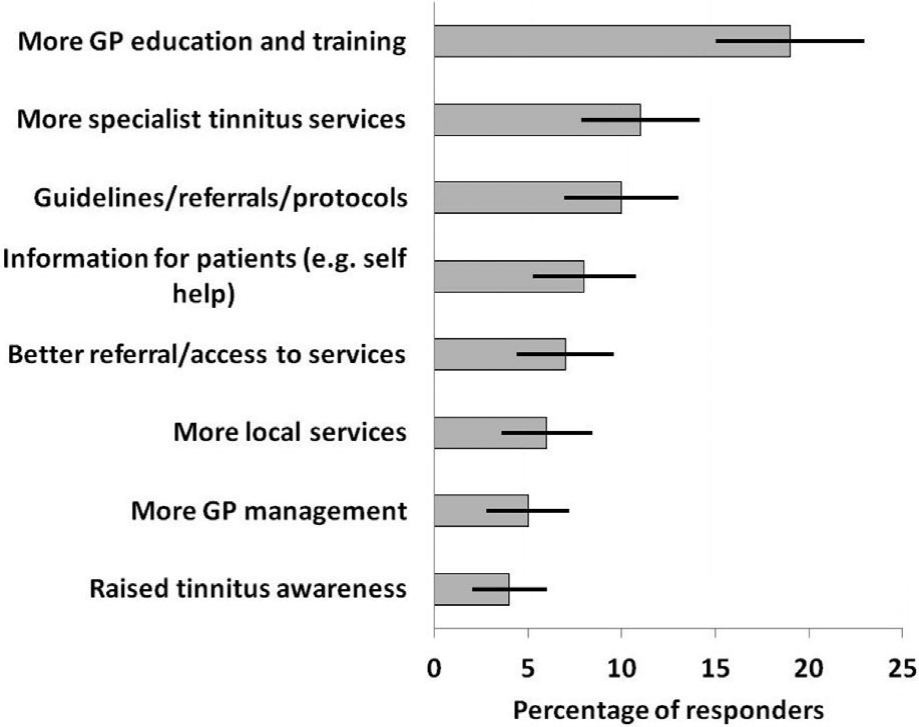
General practitioner (GP) expressed methods for improving tinnitus management in primary care. Numbers are number of GPs who indicated each response item ±95% confidence intervals.

When asked directly if tinnitus sufficiently impacted on their practice to warrant dedicated training (Appendix 1, Question 8), 28% of responders indicated that it did. Some indicated that they would approve of succinct (∼1 hour) training, either online, or as part of a broader ENT workshop. In Question 9 (Appendix 1), where GPs were invited to comment on how they felt tinnitus management in primary care could be improved, the dominant theme emerging was again a desire for concise accessible training on tinnitus management (69 responders, 19%) (Fig. 6). This viewpoint varied somewhat by SHA, from 42% in the East Midlands to 15% in South Central. Responders also suggested that increased specialist tinnitus services and easier access to those services would improve their management options.

## Discussion

### Summary of main findings

We surveyed GPs in England asking them how they currently assess and manage tinnitus patients, and how this might be improved. In terms of GPG recommended practices, a number of assessments such as otoscopy are used routinely, while others such as a tuning fork test are not. Similarly for management, almost all GPs report that they offer information and reassurance, but only one-third direct their patients towards a support group, or offer written information. This is in line with the high number of responders who reported a need for more support based approaches to management and greater informational resources. There is particular inequity in terms of drug prescription: only 4% of GPs would prescribe antidepressants for their tinnitus patients. This may reflect the lack of robust evidence for the efficacy of drug therapies for tinnitus [4], a lack of awareness of the GPG, or personal opinion and preferences for prescribing.

There was striking variability in referral data. Most referrals are to ENT, and a small number of patients are referred directly to audiology or other service. Not all symptoms outlined in the GPG that would indicate a need to refer tinnitus patients onwards are routinely assessed. Some responding GPs were unaware of the existence of any guidelines or other referral criteria. Only 14% of responding GPs, and significantly more GPs who have an interest in ENT, refer to the GPG. GPs with an interest in ENT were also less likely to refer patients onwards, were more likely to assess for ‘red flags’ such as pulsatile tinnitus, and were also more prepared to prescribe drug therapies when managing tinnitus independently.

It is important to consider why the GPG is not synonymous with practice. The target audience for the GPG included chief executives, medical and non-medical directors of health care, as well as GPs, audiologists, ENT consultants, and heads of audiology. The GPG was made available on the Department of Health and related websites, and presented at national conferences (e.g. the 2009 British Tinnitus Association and British Society of Audiology meetings), where its wide use, to inform local dialogue and decision making, was advocated. However, our previous survey of audiological services for tinnitus found considerable variability in the service delivered within and between departments, suggesting that the GPG has not resulted in a greater standardization of practice through commissioning and effective triage [3]. The limited use of some practices recommended in the GPG by some GPs may be the result of limited or ineffective dissemination. We did not, however, specifically ascertain how many GPs are unaware of the GPG or how many are aware and simply choose not to use it as a primary reference. Although there are links to the GPG from those internet sites most used by GPs (e.g. GPnotebook), these are not prominently displayed and require ‘within-site’ searching. Lack of synonymy between working practices and the GPG may also reflect a lack of personal belief in the efficacy of what is proposed. There may also be limited incentive for GPs to use this Department of Health guideline. For example, some GPs reported that tinnitus does not impact on their practice in any significant way, they rarely see tinnitus patients, or their tinnitus patients are tolerant of their tinnitus or ‘not very bothered’. Given that many responding GPs are clearly open to using a set of guidelines, and that some were unaware that there are such guidelines already available, the issue may be more one of dissemination than acceptance.

Clinical education was the main theme emerging from responses to open question responses with 76% of GPs telling us they do look for new information on tinnitus. Thirty per cent of responders felt that tinnitus impacted sufficiently on their practice to warrant a dedicated educational workshop. However, access to appropriate workshops would be variable across different SHAs and take up from individual GPs uncertain. Online material however, would be open to all and can be updated and quality assured. Indeed, when looking for information on tinnitus, responders predominantly refer to online materials. There is a need, however, to evaluate the standards of these sources of information to ensure that GPs are accessing the best and most ‘up-to-date’ information and advice.

### The strengths and limitations of this study

We concentrated resources on obtaining several hundred responses from just one single mail-out and so we did not send reminder letters that may have generated a larger overall *percentage* response rate. A higher response rate is desirable where we do not know the demographic of non-responders and so there is the potential for responder bias, for example, if we had only received responses from GPs who had an interest in ENT or who felt that tinnitus was an important issue within their practice. There was, however, no obvious bias in our responding sample. For example, only 18% of 368 responders reported having an interest in ENT, we received similar numbers of responses from GPs working in small, medium and large practices, and from GPs working between four and 10 sessions per week. Furthermore we received responses from GPs who rarely came across tinnitus in their clinic and those who frequently consulted tinnitus patients. To maximize the external validity of our sample, the confidence intervals we report here have been calculated relative to the approximate number of GPs in England (i.e. 31 000).

Our percentage response rate is directly comparable with that of a recent survey of GP tinnitus management in Northern Ireland where there was a 15% response rate (174 responses from 1154 GPs) [17]. It is also comparable with another ‘non-tinnitus’ GP survey by Huss and Röösli [18] who reported a 28% response rate. We admit that a GP survey response rate of 60% or more can be achieved by using a different strategy to that used here, that is, optimally timed reminder letters [12]. Our strategy was a single large mail-out that achieved close to its objective, that is, to get responses from 400 GPs.

### Comparisons with the previous work

There is little evaluative literature on GP management of tinnitus. In the present study, GPs rated their satisfaction with the service they provide for tinnitus patients, and their perceived patient satisfaction, around the middle of a visual analogue scale (neither very low nor very high). This is in line with the opinion of other clinicians and patients. In our recent survey of 138 audiologists and hearing therapists almost half felt that GP management needed improvements or was not effective [19]. And in a qualitative survey of 73 tinnitus patients, one-third of responders were satisfied that their GP had done all they could do, but another third said they were not satisfied with the treatment they received, citing GPs' lack of knowledge on tinnitus, or their insensitivity to the ‘burden’ of tinnitus [20]. In a more recent survey of GPs in Northern Ireland from the Royal National Institute for Deaf People, Redmond [17] found that 57% of responders had never received *any* tinnitus training, and yet 53% rated their own knowledge of tinnitus as average or below average.

Many findings from Redmond [17] are similar to those reported here. Seventy-seven per cent of responders reported that they would like to receive tinnitus training updates, compared with the 76% of responders in the current study who reportedly look for information on tinnitus. In terms of management, Redmond [17] reported similar rates of drug prescription as reported here. For example, she found that 3% of GPs would prescribe antidepressants, compared with 4% here.

To our knowledge, the only other published study of tinnitus management in general practice came from Vanniasegaram *et al*. [21] who in 1993 surveyed small clusters of GPs throughout the British Isles (UK and Ireland). They reported an average tinnitus patient referral rate of 43% (compared with 37% here). The most commonly reported treatments were advice, betahistine, and ear-wax removal, again, similar to what is reported here. Vanniasegaram *et al*. [21] noted that few tinnitus patients were referred for counselling, suggesting that this may reflect a lack of such services, or a lack of awareness of the importance of psychological intervention for tinnitus patients. Nearly 20 years on, this is still an issue [3].

### Implications for future research and clinical practice

General practitioners are the point of triage for tinnitus patients in the NHS and so should be completely fluent in the Department of Health guidelines if the GPG is to be successful. Ongoing restructuring in the commissioning of health care services makes this even more important if patients are to receive an efficient and effective NHS service for tinnitus. Responses to our survey reveal that, while many recommended assessments and examinations are routinely conducted when a patient presents with tinnitus, others are only used by a subset of GPs. A difference in how tinnitus is assessed potentially affects unequal patient access to treatment, a key issue that the Department of Health GPG aimed to address. Equity of service would require the adoption of more standardized approaches to tinnitus assessment, especially the assessment for symptoms that are ‘red flags’ and require onward referral to specialist services.

There is recognition in the GPG that greater involvement of primary care in tinnitus management may require a dedicated programme of updating, education and training. GPs also appear to agree a need for concise, accessible training on tinnitus management. There is a need firstly to assess the tinnitus-related resources that are currently available and accessed by GPs, to establish their currency and evaluate the level of evidence-based guidance they contain. It will then be essential to disseminate this information widely and accessibly to GPs.

## Appendix 1

Your Primary Care Trust: ________________________________

Do you have an interest in ENT? Yes No.

Number of patients registered at your practice: _______________

Your number of clinical sessions per week: _________________

In the last month, how many patients did you see whose primary complaint was tinnitus? _______________________________

1. What information do you obtain when taking a patient history relating to tinnitus? Please tick all that apply
  Tinnitus onset and causes Hearing loss
  Tinnitus loudness Hypersensitivity to loud sounds
  Tinnitus unilateral/bilateral Vertigo
  Tinnitus pulsatility General medical health
  Tinnitus quality (hissing, ringing, buzzing) Medication
  Impact of tinnitus on quality of life
2. When examining a patient with tinnitus what examination(s) do you routinely perform? Please tick all that apply
  Otoscopy Carotid bruit
  Cranial nerve examination Tuning fork test
  Blood pressure
  Other, please specify _________________________________
3. Please indicate which of the following tinnitus treatments you routinely use in your practice? Please tick all that apply
  Removal of ear wax
  Information leaflets provided by charities
  Advice and reassurance
  Provision of sound devices
  Direct towards self-help groups
  Drug therapies, please specify __________________________
  In-house counselling, please specify _____________________
  Other, please specify _________________________________
4. Do you follow any routine criteria for onward referral?
  Yes, please specify __________________________________
  No, please comment _________________________________
5. What proportion of all tinnitus patients do you refer onwards ___%
  Of the patients that are referred on, what proportion are referred to the following:
  A GP with more expertise __% Audiology __%
  Psychology __% ENT __%
  Psychiatry __% Other __%
6. On a scale from 0 to 10, how satisfied are you with the tinnitus management (including referral) offered by your practice? And how satisfied do you think your patients are with this service?
7. Where do you look for information on tinnitus? Please tick all that apply
  Do not look to any particular resources
  DH Good Practice Guidelines for tinnitus
  Online clinical resources, please specify _________________
  Advice from colleagues, please specify __________________
  Other, please specify _________________________________
8. Does tinnitus management have sufficient impact on your practice to warrant specific training, e.g. a training workshop?
  Yes No
  Any comments? _____________________________________
9. How might tinnitus management be improved in the primary care setting?
